# ?Two new species of the subgenus Reticularisus (Lepidoptera, Limacodidae, *Rhamnosa*) from China, with a checklist of the genus *Rhamnosa* Fixsen, 1887

**DOI:** 10.3897/zookeys.1099.76163

**Published:** 2022-05-03

**Authors:** Jun Wu, Ting-Ting Zhao, Hui-Lin Han

**Affiliations:** 1 School of Forestry, Northeast Forestry University, Harbin, 150040, China Northeast Forestry University Harbin China; 2 Key Laboratory of Sustainable Forest Ecosystem Management, Ministry of Education, Northeast Forestry University, Harbin, 150040, China Northeast Forestry University Harbin China; 3 Northeast Asia Biodiversity Research Center, Northeast Forestry University, Harbin, 150040, China Northeast Forestry University Harbin China

**Keywords:** Guangdong, Hunan, slug caterpillar moths, taxonomy, Zygaenoidea

## Abstract

Two new species of the subgenus Reticularisus Wu, Wu & Han, 2022 of the genus *Rhamnosa* Fixsen, 1887, Rhamnosa (Reticularisus) chenjuni**sp. nov.** and Rh. (R.) mangshanensis**sp. nov.**, are described from the provinces of Hunan and Guangdong, China. The adults and genital structures of the new species and similar examined species are illustrated. A checklist of the genus is provided.

## ?Introduction

The genus *Rhamnosa* Fixsen, 1887 was erected based on the type species *Rh.angulata* Fixsen, 1887 from “Korea”. Since then, approximately ten new species have been described and reported (Fixsen 1887; Hering 1931, 1933; Matsumura 1931; Okano and Pak 1964; Wu 2008; Wu and Fang 2009). Solovyev and Witt (2009) divided *Rhamnosa* into two subgenera, *Rhamnosa* Fixsen, 1887 and *Caniodes* Matsumura, 1927, based on the external features and the male genitalia. Later, Solovyev and Dubatolov (2015) clarified the exact taxonomic position of some species and provided a distribution map for *Rh.angulata* Fixsen, 1887. Solovyev (2017) established a third subgenus, *Rhamnopsis* Matsumura, 1931, for the endemic species *Rh.arizanella* (Matsumura, 1931) from Taiwan. However, *Rh.arizanella* was misplaced in the subgenus Rhamnosa by Wu et al. (2022), which is corrected in this paper. A fourth subgenus, *Reticularisus* Wu, Wu & Han, 2022, was established based on overall appearance and male genitalic characters. This subgenus contains two species: Rh. (R.) henanensis Wu, 2008 and Rh. (R.) shierbeihoua Wu, Wu & Han, 2022 (Wu et al. 2022). To date, the genus included nine described species belonging to four subgenera, all of which have been recorded in China.

In this study, two new species of the subgenus Reticularisus, Rh. (R.) chenjuni sp. nov. and Rh. (R.) mangshanensis sp. nov., collected from the Hunan and Guangdong provinces of China, are described.

## ?Materials and methods

The specimens were collected with a 220V/450W mercury vapour lamp and DC black light lamps at Mangshan National Nature Reserve and Nanling National Forest Park, respectively in the Hunan and Guangdong provinces of China. Standard methods for dissection and preparation of the genitalia slides were used (Kononenko and Han 2007). The specimens were photographed using a Nikon D700 camera, whereas the genitalia slides were photographed with an Olympus photo microscope and processed using the Helicon Focus software and Adobe Photoshop CS6. All type materials of the new species are deposited in the collection of the Northeast Forestry University (**NEFU**), Harbin, China. Material from the National Zoological Museum of China, Institute of Zoology, Chinese Academy of Sciences, Beijing, China (**IZCAS**) was also examined in this study.

## ?Taxonomic account

### ?Genus Rhamnosa

Taxon classificationAnimaliaLepidopteraLimacodidae

Fixsen, 1887

4E2D21A7-D096-5C4B-AAF7-DA0A50578331

Rhamnosa Fixsen, 1887: 339. Type species: Rhamnosaangulata Fixsen, 1887, by monotypy. Type locality: “Korea”.
Caniodes Matsumura, 1927: 91. Type species: Caniodestakamukui Matsumura, 1927. Type locality: “Formosa” (Horisha).
Rnamnopsis Matsumura, 1931: 101. Type species: Rhamnopsisarizanella Matsumura, 1931. Type locality: “Formosa” (Arisan).

### Subgenus Reticularisus

Taxon classificationAnimaliaLepidopteraLimacodidae

Wu, Wu & Han, 2022

FAAD39BA-C644-51FE-BBF8-B01B3C73068A

Reticularisus Wu, Wu & Han, 2022: 138. Type species: Rhamnosahenanensis Wu, 2008, by original designation. Type locality: Henan Province, China.

#### Notes.

The subgenus is characterized by the forewing being pale yellow in ground colour and covered with reddish-brown scales. The antemedial and postmedial lines are entire, not parallel, straight or slightly curved, darkish, running from the wing margin near the apex to the inner margin. The venation of the forewing is usually of an obvious dark brownish red colour. The species are not sexually dimorphic; the females are usually slightly larger, with filiform or slightly bipectinated antennae.

The male genitalia are diagnostic: apical part of juxta with massive numbers of tiny spines; basal part flat, with a pair of lateral processes that can be strongly sclerotized or not; saccus visible or simply present; valva without basal processes; aedeagus slender and always more or less spiral-shaped near the coecum.

### Rhamnosa (Reticularisus) chenjuni sp. nov.

Taxon classificationAnimaliaLepidopteraLimacodidae

41A397E1-4FA7-5DB8-A0A6-324CC9924FE5

http://zoobank.org/DF51CFAB-D023-45C3-8B51-A0EF7CB0D55F

Figs 1 , 2 , 7 , 11

#### Holotype.

?, China, Hunan Province, Chenzhou City, Yizhang County, Mangshan National Nature Reserve, Jiangjunzhai scenic spot, 30.VII–7.VIII.2021, leg. J. Wu and Q. Lin, genit. prep. WuJ-583-1 (NEFU).

#### Paratypes.

17?, 4?, same date as for holotype, genit. prep. WuJ-582-1, WuJ-584-2, WuJ-585-2 (NEFU). 2?, China, Guangdong Province, Shaoguan City, Ruyuan County, Nanling National Forest Park, 24–27.V.2021, leg. MR. Li and G. Fu, genit. prep. WuJ-594-1, WuJ-595-1 (NEFU).

#### Diagnosis.

**Figures 1–6.**
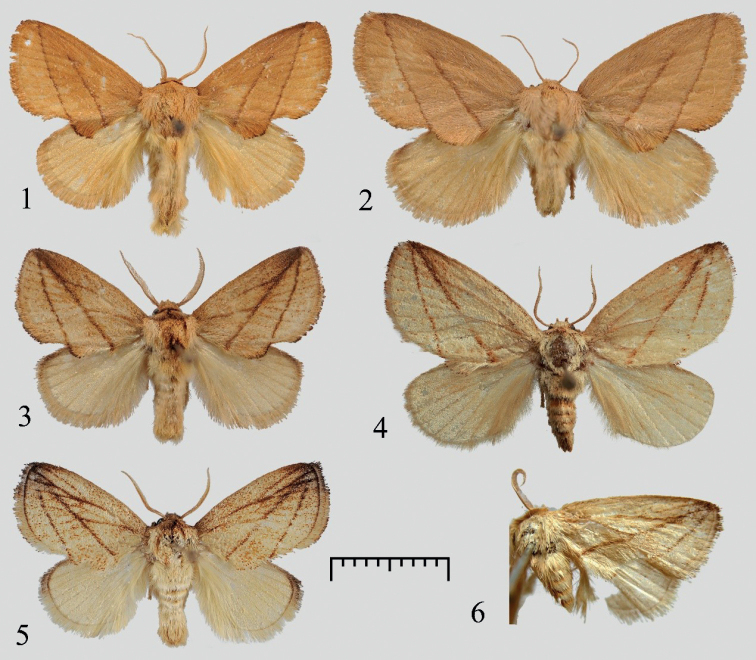
Adults of Rhamnosa (Reticularisus) spp. **1***Rh.chenjuni* sp. nov., male, holotype (NEFU) **2***Rh.chenjuni* sp. nov., female, paratype (NEFU) **3***Rh.mangshanensis* sp. nov., male, holotype (NEFU) **4***Rh.mangshanensis* sp. nov., female, paratype (NEFU) **5***Rh.shierbeihoua* Wu, Wu & Han, 2022, male, holotype, Guizhou, China (NEFU) **6***Rh.henanensis* Wu, 2008, male, holotype, Henan, China (IZCAS). Scale bar: 1 cm.

The new species *Rh.chenjuni* sp. nov. (Figs 1, 2) can be distinguished from the other three species (Figs 3–6) in the subgenus Reticularisus by the forewing patterns. The antemedial line of the forewing is barely visible in the region near the apex and does not intersect with the subterminal line, but in *Rh.mangshanensis* sp. nov. (Figs 3, 4), *Rh.shierbeihoua* (Fig. 5), and *Rh.henanensis* (Fig. 6), the antemedial lines are entire and intersect with the subterminal lines at the wing margin near the apex.

**Figures 7–12.**
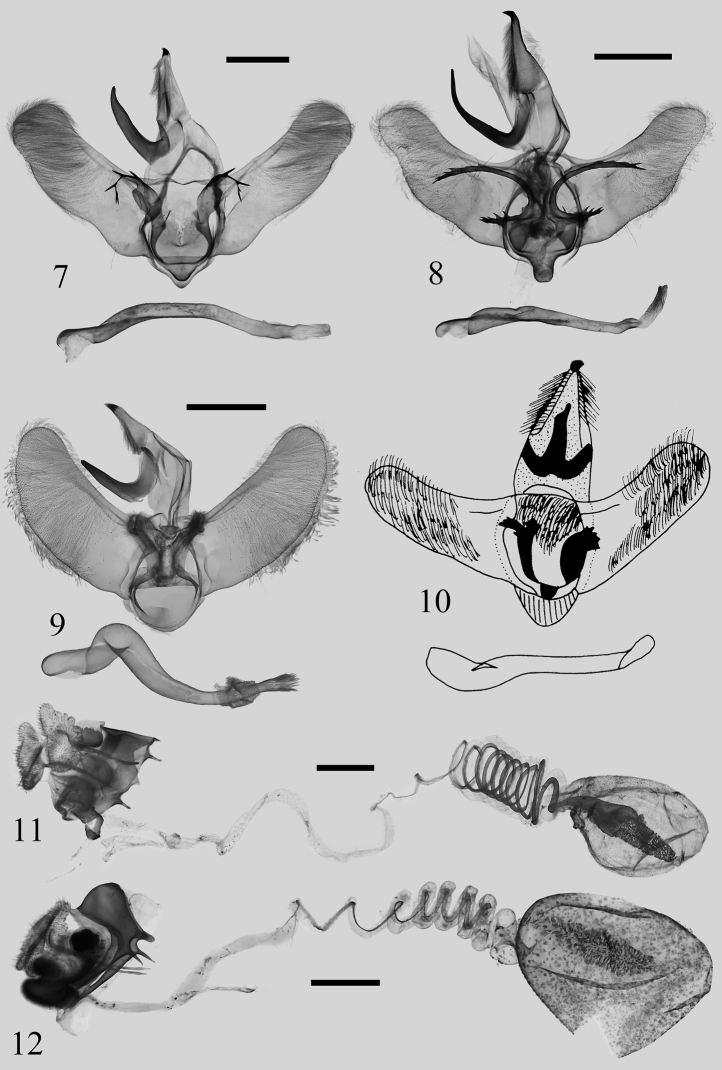
Genitalia of Rhamnosa (Reticularisus) spp. **7***Rh.chenjuni* sp. nov., male, holotype (NEFU) **8***Rh.mangshanensis* sp. nov., male, holotype (NEFU) **9***Rh.shierbeihoua* Wu, Wu & Han, 2022, male, holotype, genit. slide WuJ-301-1 (NEFU) **10***Rh.henanensis* Wu, 2008, male, holotype (IZCAS) **11***Rh.chenjuni* sp. nov., female, paratype, genit. slide WuJ-585-2 (NEFU) **12***Rh.mangshanensis* sp. nov., female, paratype, genit. slide WuJ-593-2 (NEFU). Scale bars: 1 mm.

In the male genitalia, *Rh.chenjuni* sp. nov. (Fig. 7) is most similar to the other new species *Rh.mangshanensis* sp. nov. (Fig. 8), but the diagnostic features are the short and stout gnathos, the lateral processes of the juxta strongly sclerotized and gradually diverging into 3–7 long, acuate spines, and the saccus is small and triangular rather than tongue-shaped. However, in *Rh.mangshanensis* sp. nov. the gnathos is slender and curved at the middle; the lateral processes of the juxta bear a strongly sclerotized, long, hook-shaped process, and the saccus is tongue-shaped. *Rh.chenjuni* sp. nov. differs from *Rh.shierbeihoua* (Fig. 9) and *Rh.henanensis* (Fig. 10) by the following characteristics of the male genitalia: the sacculus of the valva is wavy; the juxta bears a pair of lateral processes that are strongly sclerotized and gradually diverging into several long spines; the saccus is visible, small, triangular. However, in *Rh.shierbeihoua*, the sacculus of the valva is smoothly arc-curved, the lateral processes of juxta are short, not sclerotized, covered with massive numbers of spinules, and the saccus is not visible. In *Rh.henanensis*, the sacculus of the valva is straight, the juxta bears a pair of sawblade-shaped and strongly sclerotized lateral processes, and the saccus is short and broad.

In the female genitalia, *Rh.chenjuni* sp. nov. (Fig. 11) differs from *Rh.mangshanensis* sp. nov. (Fig. 12) by its strongly swollen genital chamber, highly modified lamella postvaginalis, less spiraled ductus bursae, larger corpus bursae, and the upper position of the signum.

#### Description.

Adult (Figs 1, 2). Forewing length 11–13 mm, wingspan 23–29 mm in male (13–15 mm and 29–34 mm in female). Head brown; labial palpus short, brown; antennae bipectinated almost to the apex in male, filiform in female. Forewing ground colour ochreous to pale brown, with two distinct, slightly sinuous, dark brown antemedial and subterminal lines running from costal margin near apex, and reaching inner margin at ca. 1/3 and 2/3 distance from the wing base, respectively; antemedial line barely visible near apical region; a conspicuous dentiform tuft located at middle of inner margin; fringe ochreous with black terminally. Hindwing pale yellow, mixed with a little brown. Scales on legs ochreous to pale yellow. Abdomen pale yellow.

**Male genitalia** (Fig. 7). Uncus triangular, with a strongly sclerotized apical spur. Gnathos hook-shaped, slightly thinner terminally. Valva of almost equal width, upper half part covered with dense hairs; sacculus obviously waved; cucullus broad and rounded. Juxta flattened, rounded, slightly divided apically; lateral process plate-shaped, bearing a strongly sclerotized process gradually diverging into 3–7 (normally of 5) long, acuate spines. Saccus visible, as a small triangle. Aedeagus slender, slightly spiral-shaped near coecum; cornuti of vesica not obvious.

**Female genitalia** (Fig. 11). Papillae anales ear-shaped, covered with dense setae on surface, margins with a dorsal and ventral lobe and several deep clefts in the middle. Postvaginal plate strongly sclerotized, nearly square. Anterior apophysis short; posterior apophysis long and slender, ca. 4× length of anterior apophysis. Ostium bursae strongly sclerotized. Ductus bursae very long, strongly spiral-shaped in basal part. Corpus bursae pear-shaped, covered with dense spines on the outside and with a spindle-shaped signum that is strongly sclerotized and almost as long as corpus bursae.

#### Distribution

**Figures 13–16.**
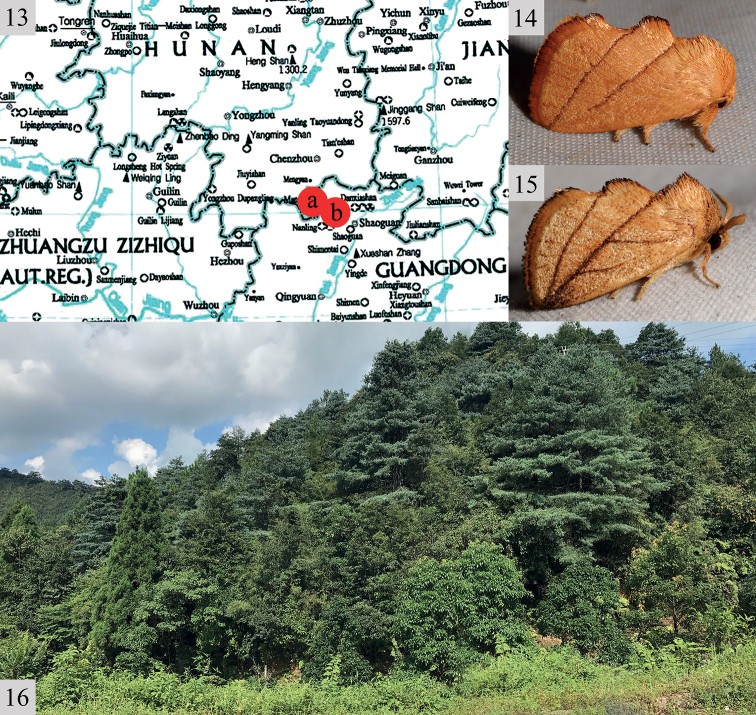
**13** collecting sites of the two new species: Hunan Province, Mangshan National Nature Reserve (red dot a); Guangdong Province, Nanling National Forest Park (red dot b) **14, 15** adult male, living habitus: **14***Rh.chenjuni* sp. nov. **15***Rh.mangshanensis* sp. nov. **16** the biotope of these two new species in Hunan is close to a mixed coniferous and broad-leaved forest.

**(Fig. 13).** China (Hunan: Mangshan; Guangdong: Nanling).

#### Etymology.

The species name is dedicated to Mr. Jun Chen, who works in the Mangshan State-owned Forestry Administration in Hunan Province, China. He was of great assistance to us when we were collecting in Mangshan National Nature Reserve.

#### Remarks.

This new species differs clearly in appearance from the other three species in the subgenus Reticularisus, mainly in having antemedial line not visible near the apical region and forewing lacking distinctive marks other than the antemedial and submarginal lines. It shares some similarities with Rh. (Rhamnosa) hatita (Druce, 1896); however, because it highly matches the characters of the subgenus Reticularisus for the male genitalia, i.e., valva without a basal process, juxta with a pair of distinct lateral processes, saccus visible, and aedeagus spiraled near coecum, it is provisionally placed in this subgenus.

These moths fly from late May to August. The specimens were collected by 220V/450W mercury light and DC black light at 570–1,265 m a.s.l.; the collecting site in Hunan province is located close to mixed coniferous and broad-leaved forests (Figs 14, 16).

### Rhamnosa (Reticularisus) mangshanensis sp. nov.

Taxon classificationAnimaliaLepidopteraLimacodidae

803EDA45-109B-5793-95A3-2A7A879A8C43

http://zoobank.org/72F98AFB-557A-49B0-BB6A-68FAB1D4009F

Figs 3 , 4 , 8 , 12

#### Holotype.

?, China, Hunan Province, Chenzhou City, Yizhang County, Mangshan National Nature Reserve, Jiangjunzhai scenic spot, 30.VII–7.VIII.2021, leg. J. Wu and Q. Lin, genit. prep. WuJ-581-1 (NEFU).

#### Paratypes.

27?, same date as for holotype, genit. prep. WuJ-579-1, WuJ-580-1 (NEFU); 1?, 1?, China, Guangdong Province, Shaoguan City, Ruyuan County, Nanling National Forest Park, 24–27.V.2021, leg. MR. Li and G. Fu, genit. prep. WuJ-592-1, WuJ-593-2 (NEFU).

#### Diagnosis.

Three of the species in the subgenus Reticularisus, *Rh.mangshanensis* sp. nov. (Figs 3, 4), *Rhamnosa.shierbeihoua* (Fig. 5), and *Rh.henanensis* (Fig. 6), are very similar in appearance. *Rh.mangshanensis* sp. nov. can be distinguished from *Rh.shierbeihoua* by the point of emergence of the two oblique antemedial and subterminal lines (running from the costal margin near the apex in *Rh.mangshanensis* sp. nov. but from the outer margin near the apex in *Rh.shierbeihoua*), and by the ground colour of the body (pale brownish-yellow in *Rh.mangshanensis* sp. nov. but pale yellow in *Rh.shierbeihoua*). However, it is hard to distinguish it from the *Rh.henanensis* only by its external appearance.

The male genitalia of *Rh.mangshanensis* sp. nov. (Fig. 8) are clearly distinguishable from those of *Rh.shierbeihoua* (Fig. 9) and *Rh.henanensis* (Fig. 10). The gnathos of *Rh.mangshanensis* sp. nov. is slender and up-curved at an obtuse angle at the middle, the sacculus of the valva is distinctly waved, and the lateral processes of the juxta are strongly sclerotized with a long, slender, hook-shaped, basally serrated and terminally forked process. In *Rh.shierbeihoua* (Fig. 9) and *Rh.henanensis* (Fig. 10) the gnathos are shorter and thicker, the sacculi are straight or smoothly arc-curved, the lateral processes of the juxta are short, nearly plate-shaped, without a long slender process at apex. The differences in external appearance and genitalia between *Rh.mangshanensis* sp. nov. and *Rh.chenjuni* sp. nov. are listed under the latter species.

#### Description.

Adult (Figs 3, 4). Forewing length 11–12 mm, wingspan 24–27 mm in male (14 mm and 29 mm in the single studied female). Head dark brown; labial palpus short, dark brown; male antennae bipectinated almost to apex, female antennae also bipectinated but extremely thinner than male’s. Thorax brownish yellow; patagium reddish-brown; tegula brownish-yellow. Forewing ground colour pale brownish-yellow; costal margin dark brown to black near apex; two distinct, oblique, dark brown antemedial and subterminal lines running from costal margin near apex to inner margin: antemedial line straight, reaching to ca. 1/3 from wing base, subterminal line slightly curved towards outer margin, reaching to ca. 2/3 from wing base; a mixed brownish-yellow and dark brown dentiform tuft is located between these two lines along the inner margin; venation visible in forewing, brown, veins at margins of cell dark brown; fringe dark brown to black. Hindwing pale yellow; fringe dark brown at apex, remainder pale yellow. Scales on legs brown to pale yellow. Abdomen brownish-yellow.

**Male genitalia** (Fig. 8). Uncus triangular, elongated, with a strongly sclerotized apical spur. Gnathos slender, hook-shaped, up-curved at an obtuse angle at middle. Valva broad at base; sacculus obviously waved; cucullus slightly narrower, rounded. Juxta highly modified, covered with massive numbers of tiny spines in upper part; basal part with a pair of strongly sclerotized, long, slender, hook-shaped lateral processes, with a row of teeth at base and terminally forked. Vinculum narrow. Saccus strongly sclerotized, tongue-shaped. Aedeagus slender, slightly spiral-shaped near coecum, sclerotized at apex; vesica with dense, tiny cornuti.

**Female genitalia** (Fig. 12). Papillae anales ear-shaped, covered with dense setae on surface, margins with several small clefts. Anterior apophysis short but robust, pointed apically, with an obvious tongue-shaped process next to it; posterior apophysis long and slender, slightly enlarged subapically, ca. 4.5× length of anterior apophysis. Genital chamber strongly sclerotized and obviously swollen, with a pair of rounded processes below it. Lamella postvaginalis highly modified, oval-shaped, densely covered with short hairs, with a pair of small hairy processes. Ductus bursae long, membranous, thick and strongly spiral-shaped at base. Corpus bursae large, oval-shaped, densely covered with tiny sclerotized flecks, with a spindle-shaped, strongly sclerotized, erect signum situated in upper 2/3.

#### Distribution

**(Fig. 13).** China (Hunan: Mangshan; Guangdong: Nanling).

#### Etymology.

The new species is named after its type locality, the Mangshan National Nature Reserve of Hunan Province, China.

#### Remarks.

These moths fly from late May to August. The specimens were collected by 220V/450W mercury light and DC black light at 570–1,265 m a.s.l.; the collecting site in Hunan province is close to mixed coniferous and broad-leaved forests (Figs 15, 16).

## ?Checklist of species of the genus *Rhamnosa* Fixsen, 1887

**Subgenus Rhamnosa Fixsen, 1887**

Rh. (R.) angulata Fixsen, 1887

Rh. (R.) hatita (Druce, 1896)

= *Rh.angulatekwangtungensis* Hering, 1931

= Rh. (R.) kwangtungensis Hering, 1931

Rh. (R.) dentifera Hering & Hopp, 1927

Rh. (R.) convergens Hering, 1931

**Subgenus Caniodes Matsumura, 1927**

Rh. (C.) uniformis (Swinhoe, 1895)

= *Canianotodonta* Hampson, 1897

= *Caniodestakamukui* Matsumura, 1927

= *Rh.uniformisrufina* Hering, 1931

Rh. (C.) uniformoides Wu & Fang, 2009

**Subgenus Rhamnopsis Matsumura, 1931**

Rh. (R.) arizanella (Matsumura, 1931)

= *Rhamnopsisarizanella* Matsumura, 1931

= *Rh.arizanella* (Matsumura, 1931)

**Subgenus Reticularisus Wu, Wu & Han, 2022**

Rh. (R.) henanensis Wu, 2008

Rh. (R.) chenjuni sp. nov.

Rh. (R.) mangshanensis sp. nov.

Rh. (R.) shierbeihoua Wu, Wu & Han, 2022

## ?Discussion

The genus *Rhamnosa* includes four subgenera with a total of eleven species, all 11 species are recorded in China based on the literature. The subgenus Reticularisus was established for its unique characteristics of the forewing and male genitalia. In the four known species of the subgenus, the antemedial and subterminal lines of the forewing always intersect near the apex (the antemedial line of Rh. (R.) chenjuni sp. nov. is not visible anteriorly so the two lines do not intersect, but the two lines would cross if both visible), and the aedeagus is always more or less spiral-shaped near the coecum, so these two characters may be considered as apomorphies of the subgenus.

## Supplementary Material

XML Treatment for Rhamnosa

XML Treatment for Subgenus Reticularisus

XML Treatment for Rhamnosa (Reticularisus) chenjuni

XML Treatment for Rhamnosa (Reticularisus) mangshanensis
